# Financial Toxicity of Hematologic Malignancy Therapies, Including Cellular Therapy and Its Impact on Access to Care: Prospective Pilot Study

**DOI:** 10.2196/68101

**Published:** 2026-01-06

**Authors:** Lemchukwu Amaeshi, Lauren Laufer, Kateryna Fedorov, Alex Sisto, David Levitz, Rosmi Mathew, Karen Wright, Dennis Cooper, Mendel Goldfinger, Ioannis Mantzaris, R Alejandro Sica, Kira Gritsman, Marina Konopleva, Ridhi Gupta, Noah Kornblum, Chenxin Zhang, Mimi Kim, Amit Verma, Bruce Rapkin, Aditi Shastri, Nishi Shah

**Affiliations:** 1 Department of Medicine, Montefiore Medical Center, Bronx, NY, United States; 2 Department of Medicine, Albert Einstein College of Medicine, Bronx, NY, United States; 3 Department of Medicine, University of California, Los Angeles, CA, United States; 4 Department of Oncology, Montefiore Medical Center and Albert Einstein College of Medicine, 111 E 210th St, Bronx, NY, 10467, United States, +1 (404) 778-1900; 5 Oncology, Vanderbilt University Medical Center, Nashville, TN, United States; 6 Department of Internal Medicine, Rutgers Robert Wood Johnson Medical School, New Brunswick, NJ, United States; 7 Department of Leukemia, The University of Texas MD Anderson Cancer Center, Houston, TX, United States

**Keywords:** financial toxicity, hematologic malignancies, cancer, cellular therapy, quality of life, socioeconomically, longitudinal study, questionnaire, cancer care, African American, Hispanic, toxicity, survival rates, survival, medical care, adherence, financial literacy

## Abstract

**Background:**

Patients with cancer often face significant financial challenges, known as financial toxicity (FT), which is associated with reduced quality of life. Patients with hematologic malignancies (HMs) are especially vulnerable due to intensive and prolonged treatments, frequent hospital visits, and a high risk of complications. While FT affects many in the general population, it is particularly severe among racial and ethnic minorities, especially those below the poverty line. To our knowledge, no studies have specifically examined FT in this vulnerable group in the United States.

**Objective:**

This study aimed to evaluate the severity of FT in patients receiving treatment for HMs in a socioeconomically underserved population, explore sociodemographic factors that may predict the severity of FT, and evaluate the subjective experiences of these patients as they relate to FT.

**Methods:**

We conducted a prospective, observational, longitudinal study at the Montefiore Cancer Center’s outpatient department in the Bronx, New York, from October 1, 2022, to October 30, 2023. Participants included either adult patients newly diagnosed (ND) with HMs or those already diagnosed, undergoing cellular therapy (CT). The severity of FT was assessed using the validated Comprehensive Score for Financial Toxicity–Functional Assessment of Chronic Illness Therapy (COST-FACIT) questionnaire. Additionally, an investigator-designed questionnaire was developed to gather sociodemographic data and evaluate the subjective effects of financial burden on patient care. Patients in both the ND and CT groups were followed for 90 days. Data collection occurred at their initial presentation, as well as on days 30 and 90.

**Results:**

Ninety patients participated in the study (ND=52 and CT=38). The median age was 59 (IQR 44-66) years, with 27% (n=24) African American and 55% (n=48) Hispanic. Overall, 75% (n=67) of participants experienced some degree of FT, most with mild FT at baseline (day 0, median COST-FACIT score=19.4). In the CT group, FT worsened significantly over time, with a decline in median COST-FACIT scores from 19.9 at day 0 to 15.5 on day 90 (*P*=.02). In a multivariable linear regression model, race and ethnicity were a significant predictor of FT burden: identifying as African American or Hispanic was associated with a significantly lower COST-FACIT score (ie, higher FT) compared to non-Hispanic White participants (B=−3.08, *P*=.04, 95% CI −6.05 to −0.12). Additionally, over half of ND and CT participants reported difficulty affording basic necessities (ND: 28/52, 54%; CT: 23/38, 61%) and concerns regarding transportation access and costs (ND: 26/50, 52%; CT: n=18/38, 47%).

**Conclusions:**

FT is prevalent among patients with HMs receiving care in underserved populations, and the burden is significantly higher among African American and Hispanic populations.

## Introduction

Cancer continues to be a significant public health concern in the United States. According to the American Cancer Society, in 2022, there were nearly 2 million new cancer cases and over 600,000 cancer-related deaths in the United States [1]. Receiving a new cancer diagnosis brings untold physical, emotional, and psychological distress to the patient and their caregivers. Although survival rates of patients with cancer have improved over the years due to advances in diagnosis and treatment, the rising cost of cancer care has become a significant challenge for patients and their providers in the US health care system. Financial toxicity (FT) has emerged in oncologic care to describe the psychological, material, and behavioral hardships arising from the economic burden of cancer [2].

Indeed, several studies have reported a close association between FT and reduced quality of life, delays in seeking medical care, nonadherence with treatment, emotional and psychological distress, and reduced overall survival in patients with cancer [3-5]. The impact of FT is particularly pronounced in patients with hematologic malignancies (HMs) as they must deal with the high cost of therapy, especially with the shift from conventional chemotherapy to immunotherapy, multiple infusion visits, prolonged hospitalizations due to life-threatening presentations, long duration of intensive treatment, and treatment-related complications [6,7]. In 2014, the average cumulative costs of hematologic cancer care in the United States ranged from approximately US $200,000 for chronic leukemias to greater than US $800,000 for acute leukemias within the first 3 years of treatment. In comparison, the cost for lung cancer was around US $250,000 and that for colorectal cancer was approximately US $150,000 [8].

The severity of FT is also determined by the patient’s sociodemographic and socioeconomic factors. Extremes of age, Black race, lower income level, limited ability to provide basic household needs, unemployment, and insurance status are associated with worse FT [2,9-11]. The factors associated with worse FT are predominant in households living below the federal poverty line. This population primarily comprises Native American, Black, and Hispanic individuals [11].

Despite growing attention to FT, most studies were conducted predominantly on the White population, who are often insured. To the best of our knowledge, no similar studies in the United States have specifically targeted racial and ethnic minorities with HM in underserved areas in the United States. This study aims to assess the severity of FT, examine relevant sociodemographic factors influencing FT, and explore the subjective experience of FT among patients with HMs.

This study was conducted in a large academic hospital in the Bronx, where more than 25% of the population lives below the federal poverty line [12].

## Methods

### Study Design and Setting

This prospective observational study was conducted over 12 months, from October 1, 2022, to October 30, 2023, at Montefiore Medical Center in Bronx, New York. The study took place in both outpatient and inpatient hematologic oncologic units of the Montefiore Cancer Center, a quaternary academic center serving a predominantly low-income, racially and ethnically diverse population.

### Participant Eligibility and Recruitment

Eligible participants included adults over 18 years old with a HM (ie, acute or chronic leukemia, Hodgkin lymphoma, non-Hodgkin lymphoma, or multiple myeloma) who either received a new diagnosis or were being evaluated for cellular therapy (CT), such as autologous or allogeneic stem cell transplantation (SCT) or chimeric antigen receptor T-cell (CAR-T) therapy.

Patients were excluded if (1) they were being seen for conditions other than HMs, (2) they were seeking a second opinion after treatment at a different institution, (3) they presented after recurrence ineligible for autologous or allogeneic SCT and CAR-T therapy, (4) they were being evaluated for a second autologous SCT as part of tandem autologous transplantation or for recurrent myeloma, or (5) they lacked capacity or were non-English or non-Spanish speakers.

Recruitment took place during routine clinical visits and on the oncology floors. Eligible patients were identified by treating providers or study personnel and were invited to participate on a rolling basis. Participants were divided into two cohorts: (1) newly diagnosed (ND) and (2) undergoing CT (SCT or CAR-T).

### Data Collection and Measurements

Data were collected in person at three time points: baseline (day 0), day 30, and day 90. At each time point, the participants completed two instruments:

1. Sociodemographic and Subjective Impact Questionnaire: The research team developed an investigator-designed, bilingual (English and Spanish) questionnaire to gather information on demographics (age, sex, race and ethnicity, income, education, employment, and insurance), cancer type, and subjective experience of financial burden, including effects on basic needs and access to care.
2. Comprehensive Score for Financial Toxicity–Functional Assessment of Chronic Illness Therapy: FT was assessed using the validated Comprehensive Score for Financial Toxicity–Functional Assessment of Chronic Illness Therapy (COST-FACIT) tool [13]. It is categorized into grades 0 to 4, based on the level of FT severity, with scores ranging from 0 to 44. A score of 0 represents grade 0 or *severe toxicity*; 1‐13, grade 2 or *moderate toxicity*; 14‐25, grade 3 or *mild toxicity*; and >25, grade 4 or *no toxicity*. Hence, higher scores indicated less severe FT. The patients were assessed at three different time points: day 0, the initial visit time, day 30, and day 90.

### Statistical Analysis

Data analysis was performed using SPSS version 29 (IBM Corp). Descriptive statistics summarized demographic characteristics and COST-FACIT scores. Frequencies and percentages were calculated for categorical variables, while medians and IQRs were used for continuous variables. Comparison of the severity of FT across the timelines was determined using the Friedman test. The Mann-Whitney *U* test and Kruskal-Wallis *H* test were used to examine significant differences in FT with respect to sociodemographic groups. Linear regression analysis identified sociodemographic predictors of FT. A two-tailed *P* value of <.05 was considered statistically significant. No adjustment for multiple comparisons was made due to the exploratory nature of the study.

### Ethical Considerations

#### Study Approval

This study was conducted in accordance with ethical standards and received approval from the IRB at Albert Einstein College of Medicine and Montefiore Medical Center (approval number: IRB 2022‐13798, approval date: 09/13/2022). The research involved human participants and adhered to the principles outlined in the Declaration of Helsinki.

#### Informed Consent

The provider (MD or nursing practitioner) or study personnel obtained informed consent during the initial visit at the HM clinic or for auto/allo SCT and CAR-T evaluation. All participants gave written informed consent before enrollment. The consent process included explaining the study’s objectives, procedures, risks, and benefits and the fact that participation is voluntary. For patients unable to give written consent, verbal consent was obtained in the presence of a trained research coordinator, per institutional policy.

#### Privacy and Confidentiality

All collected data were deidentified prior to analysis to protect participant confidentiality. Study data were stored in secure, password-protected databases accessible only to authorized personnel. No identifiable personal health information was used in any publication or presentation.

#### Compensation

Participants were not financially compensated for their involvement in this study.

## Results

### Sociodemographic Characteristics

Over a period of 12 months, we recruited 90 patients who met eligibility criteria and consented to the study. The sociodemographic characteristics of these participants are shown in Table 1. Fifty-two of 90 (57%) patients were ND, whereas the rest (n=38, 43%) were either currently receiving or preparing to receive CT. The median age was 59 (IQR 44-66) years. There were more male patients (n=56, 62%), 24 (27%) patients were African American, and more than half (n=48, 55%) were Hispanic. Over 60% (n=55, 63%) of the participants were not employed, and over a third (n=33, 37.1%) were on Medicaid. Regarding HMs, most patients had plasma cell dyscrasias (n=33, 37%) compared to other HMs.

**Table 1.** Sociodemographic characteristics.

Sociodemographic characteristics	Newly diagnosed group (n=52)	Cellular therapy group (n=38)	Total (n=90)
Age, median (IQR)	59 (42.5‐66.0)	58 (50.0‐67.0)	59 (43.5‐66.0)
Sex, n (%)			
Male	31 (60)	25 (66)	56 (62)
Female	21 (40)	13 (34)	34 (38)
Race, n (%)			
African American	15 (29)	8 (22)	24 (27)
Non-Hispanic White	6 (11)	8 (22)	14 (16)
Others	31 (60)	22 (56)	51 (57)
Ethnicity, n (%)			
Hispanic	28 (54)	20 (57)	48 (55)
Non-Hispanic	24 (46)	15 (43)	40 (45)
Employment status, n (%)			
Employed	21 (41)	11 (31)	32 (37)
Retired	13 (25)	9 (25)	22 (25)
On disability	9 (18)	8 (22)	17 (20)
Unemployed	8 (16)	8 (22)	16 (18)
Health insurance, n (%)			
Private	22 (43)	16 (42)	38 (43)
Medicare	10 (20)	8 (21)	18 (20)
Medicaid	19 (37)	14 (37)	33 (37)
Type of hematologic malignancy, n (%)			
Leukemias	21 (41)	10 (27)	31 (34)
Lymphomas	16 (31)	9 (24)	26 (29)
Plasma cell dyscrasias	14 (28)	18 (49)	33 (37)

### Severity of Financial Toxicity Across Time

#### Overview

**Figure 1.**
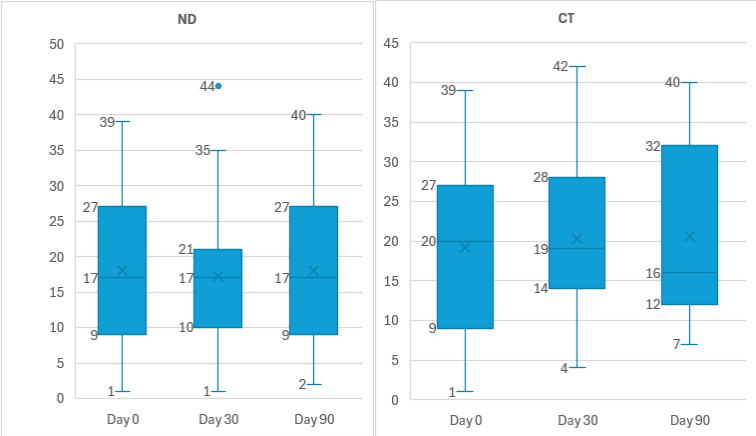
Box-plot graph comparing COST-FACIT scores across the timelines for both groups. COST-FACIT: Comprehensive Score for Financial Toxicity–Functional Assessment of Chronic Illness Therapy; CT: cellular therapy; FT: financial toxicity; FT 0: FT score day 0; FT 30: FT score day 30; FT 90: FT score day 90; ND: newly diagnosed.

Table 2 and Figure 1 depict the median FT scores at various periods. Over 75% (n=67) of patients experienced some degree of FT, with a median COST score of 19.4 at baseline (day 0). While the median COST score indicates mild FT, many patients experienced moderate FT at the individual level. The CT group demonstrated significant changes in FT scores over time (day 0: 19.9; day 30: 19.0; day 90: 15.5; *P*=.02). When comparing FT severity between the ND and CT groups, as shown in Table 3, no statistically significant differences were observed at any period (day 0: *P*=.88; day 30: *P*=.54; day 90: *P*=.75).

**Table 2.** Financial toxicity at days 0, 30, and 90.

Financial toxicity	Day 0	Day 30	Day 90	*P* value
Newly diagnosed group				
Number of patients, n	50	39	32	
Median FT^a^ (IQR)	16.5 (8.9-27.5)	17.0 (9.5-21.0)	17.5 (9.0-27.0)	.85
Grade category n (%)				
No toxicity	11 (22)	7 (18)	9 (28)	
Mild toxicity	17 (34)	18 (46)	10 (31)	
Moderate toxicity	21 (42)	14 (36)	13 (41)	
Severe toxicity	1 (2)	0 (0)	0 (0)	
Cellular therapy group				
Number of patients, n	36	29	16	
Median FT (IQR)	19.9 (9.0-27.0)	19.0 (13.8-28.0)	15.5 (12.3-32.3)	.02
Grade category n (%)				
No toxicity	10 (28)	8 (28)	5 (31)	
Mild toxicity	11 (31)	14 (48)	4 (25)	
Moderate toxicity	15 (42)	7 (24)	5 (31)	
Severe toxicity	0 (0)	0 (0)	0 (0)	
Total population				
Number of patients, n	88	69	48	
Median FT (IQR)	19.4 (8.9-27.0)	18 (10.0-25.0)	17 (10.0-17.5)	.31
Grade category n (%)				
No toxicity	21 (24)	15 (22)	14 (30)	
Mild toxicity	29 (33)	33 (48)	14 (30)	
Moderate toxicity	36 (41)	21 (30)	18 (39)	
Severe toxicity	2 (2)	0 (0)	0 (0)	

a FT: financial toxicity.

**Table 3.** Comparison of financial toxicity between newly diagnosed and cellular therapy groups on days 0, 30, and 90.

Days	ND^a^	CT^b^	*P* value
Day 0
Number of patients, n	50	37
Median FT^c^ (IQR)	16.5 (8.9-27.5)	19.9 (9.0-27.0)	.88
Day 30			
Number of patients	36	32
Median FT (IQR)	17.0 (9.5-21.0)	19.0 (13.8-28.0)	.54
Day 90			
Number of patients	31	15
Median FT (IQR)	17.5 (9.0-27.0)	15.5 (12.3-32.3)	.75

a ND: newly diagnosed.

b CT: cellular therapy.

c FT: financial toxicity.

#### Financial Toxicity and Sociodemographic Factors

Table 4 summarizes the difference in FT by race and ethnicity, diagnosis, and employment status at day 0, day 30, and day 90. We examined the association between FT and sociodemographic factors such as race, ethnicity, type of HM, and employment status (employment status was recategorized as employed vs unemployed for ease of analysis). Differences in FT across racial groups were marginally significant, with White patients experiencing relatively milder FT at day 0 (median FT: 27.0 for White patients, 16.5 for African American patients, and 14.0 for other racial groups; *P*=.08) and at day 90 (median FT: 37.0 for White patients, 13.5 for African American patients, and 15.0 for other racial groups; *P*=.06).

**Table 4.** Difference in financial toxicity on days 0, 30, and 90.

	Financial toxicity, median (IQR)		
Sociodemographics	Day 0	Day 30	Day 90
Total cohort			
Race			
African American	16.5 (8.8-25.2)	18.0 (10.0-25.8)	13.5 (6.0-26.0)
White	27.0 (22.0-36.0)	20.6 (17.0-30.8)	37.0 (19.0-41.0)
Other	14.0 (8.9-24.1)	16.8 (10.5-21.2)	15.0 (10.0-27.5)
*P* value	.08	.38	.06
Ethnicity			
Hispanic	13.5 (8.9-24.8)	15.5 (8.9-21.2)	14.5 (9.8-28.0)
Non-Hispanic	20.0 (8.5-27.5)	18.0 (14.0-26.5)	19.0 (7.8-31.5)
*P* value	.45	.17	.49
Diagnosis			
Leukemia	19.4 (10.3-24.5)	18.0 (17.0-22.0)	17.0 (8.8-25.8)
Lymphoma	10.0 (8.0-28.0)	14.4 (8.4-17.6)	15.0 (4.5-27.8)
Plasma cell dyscrasias	20.0 (8.8-28.0)	19.5 (9.8-28.2)	18.0 (10.0-30.0)
*P* value	.75	.11	.77
Employment status			
Employed	12.0 (8.0-24.0)	19.0 (17.0-22.0)	17.0 (8.0-23.0)
Unemployed	15.0 (10.0-28.0)	18.0 (10.0-28.0)	15.0 (9.0-28.0)
*P* value	.13	.67	.94
Newly diagnosed cohort			
Race			
African American	15.4 (8.2-20.4)	18.0 (9.0-22.5)	13.5 (6.0-22.0)
White	33.5 (26.7-36.0)	19.8 (18.4-31.9)	22.0 (16.0-40.0)
Other	14.0 (8.8-24.2)	16.5 (9.0-20.0)	19.0 (10.0-28.0)
*P* value	.04	.46	.16
Ethnicity			
Hispanic	14.0 (9.0-28.0)	15.0 (9.0-20.0)	21.5 (10.0-28.0)
Non-Hispanic	16.5 (7.9-25.5)	18.0 (15.5-23.8)	18.0 (9.0-24.0)
*P* value	>.99	.29	.38
Diagnosis			
Leukemia	17.0 (8.0-23.0)	18.0 (15.8-20.0)	19.5 (8.5-27.3)
Lymphoma	9.8 (9.0-27.5)	12.0 (8.9-17.3)	11.0 (4.0-27.0)
Plasma cell dyscrasias	22.0 (9.1-28.0)	19.4 (8.0-28.8)	19.0 (10.0-28.0)
*P* value	.92	.29	.48
Employment status			
Employed	11.0 (7.25-26.0)	18.0 (13.3-20.5)	18.0 (9.0-22.3)
Unemployed	15.0 (9.0-15.0)	12.0 (8.0-28.0)	12.0 (6.0-28.0)
*P* value	.41	.95	.71
Cellular therapy cohort			
Race			
African American	19.5 (8.8-28.3)	24.0 (14.5-29.2)	33.0 (7.0-44.0)
White	22.0 (13.8-29.5)	22.8 (10.9-29.4)	39.5 (37.0-42)
Other	19.9 (10.0-24.0)	18.0 (13.9-1.5)	13.0 (8.75-16.25)
*P* value	.77	.55	<.001
Ethnicity			
Hispanic	16.0 (8.9-22.5)	16.0 (11.3-21.5)	13.0 (8.75-16.3)
Non-Hispanic	24.5 (9.5-31.0)	22.5 (14.5-30.9)	37.0 (20.0-43.0)
*P* value	.29	.24	.05
Diagnosis			
Leukemia	20.0 (13.0-25.0)	22.0 (19.5-29.5)	13.5 (8.75-25.5)
Lymphoma	13.0 (8.0-28.0)	15.0 (10.7-17.0)	N/A
Plasma cell dyscrasias	19.9 (8.7-27.8)	20.0 (13.5-26.8)	14.0 (8.25-32.25)
*P* value	.94	.21	.95
Employment status			
Employed	20.0 (5.0-20.0)	22.0 (12.0-29.5)	12.0 (7.5-31.5)
Unemployed	16.5 (10.3-34.0)	20.5 (15.0-29.0)	15.5 (13.3-32.8)
*P* value	.25	.45	.66

Within the ND cohort, White patients had significantly less severe FT than other racial groups at day 0 (median FT: 33.5 for White patients, 15.4 for African American patients, and 14.0 for other racial groups; *P*=.04). However, the significance was not maintained at day 30 or day 90.

A similar trend was observed in the CT group at day 90 (median FT: 39.5 for White patients, 33.0 for African American patients, and 13.0 for other racial groups; *P*<.001).

### Predictors of Financial Toxicity

A linear regression analysis was performed (Table 5) to identify potential predictors of FT, as measured by the continuous COST-FACIT score. The model incorporated sex, race and ethnicity, insurance status, employment, and type of malignancy as independent variables. Among individual predictors, race and ethnicity were the only statistically significant predictors, which were associated with a 3.08-point lower COST-FACIT score, indicating higher FT in this group (B=−3.08, *P*=.04, 95% CI −6.047 to −0.121). The other variables—sex, insurance status, employment, and malignancy type—were not significantly correlated with FT.

**Table 5.** Linear regression predicting financial toxicity using the COST-FACIT^a^ score.

Predictor	B (95% CI)	SE	β	*t*	*P* value
Sex	3.521 (−1.136 to 8.177)	2.337	.169	1.507	.14
Race/ethnicity	−3.084 (−6.047 to −0.121)	1.487	−.232	−2.074	.04
Health insurance	0.357 (−2.697 to 3.411)	1.533	.027	0.233	.82
Type of malignancy	0.185 (−2.711 to 3.080)	1.453	.014	0.127	.90
Employment	3.724 (−2.711 to 3.080)	2.511	.173	0.127	.90
Intercept	14.317 (−1.272 to 29.906)	7.824	—	1.830	.07

a COST-FACIT: Comprehensive Score for Financial Toxicity–Functional Assessment of Chronic Illness Therapy.

### Subjective Experience of Financial Toxicity

**Figure 2.**
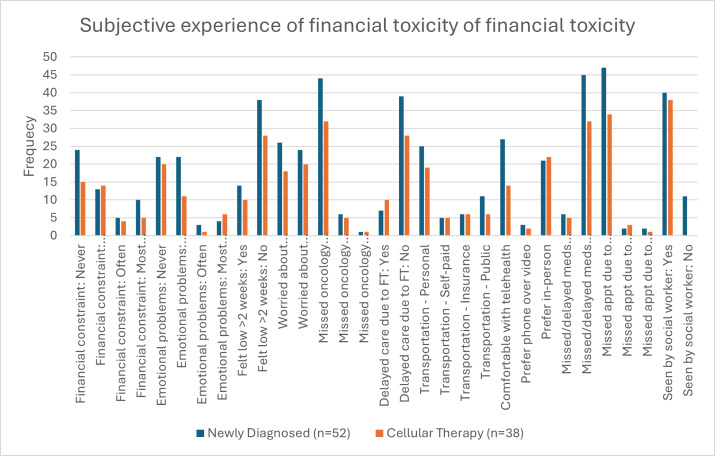
Box-plot graph showing frequencies of parameters assessing subjective experience from financial toxicity by treatment group. CT: cellular therapy; ND: newly diagnosed.

Our social workers evaluated more than 75% of the patients in our survey at the time of diagnosis. Over a third (n=32, 37%) of our patients were employed, while more than 60% were retired, disabled, or unemployed (Table 1). Table 6 and Figure 2 show the results of our investigator-based questionnaire. Most patients reported financial difficulties in carrying out their daily activities, such as paying for food, heating/air-conditioning, or warm clothes over the past 6 months (ND group: n=28, 54%; CT group: n=23, 61%). Additionally, over half of the patients experienced some degree of emotional distress in their daily lives (ND group: n=29, 57%; CT group: n=18, 48%). About 15% in the ND group and 26% in the CT group delayed seeking medical care due to financial constraints, and only about 10% missed appointments due to caregiver issues.

**Table 6.** Subjective experience of financial toxicity on patient’s well-being and access to care.

	Day 0	
Economic data	Newly diagnosed (n=52)	Cellular therapy (n=38)
Have you felt financially constrained, such as paying for food, heating/cooling in the last 6 months? n (%)		
Never	24 (46)	15 (39)
Sometimes	13 (25)	14 (37)
Often	5 (10)	4 (11)
Most of the time	10 (19)	5 (13)
During the past 4 wk, have you had any problems with your work or daily life due to any emotional problems, such as feeling depressed, sad, or anxious? n (%)		
Never	22 (43)	20 (52)
Sometimes	22 (43)	11 (29)
Often	3 (6)	1 (3)
Most of the time	4 (8)	6 (16)
Over the last 6 months, have you felt particularly low for more than 2 wk? n (%)		
Yes	14 (27)	10 (26)
No	38 (73)	28 (74)
Are you worried about access and cost of transportation for medical appointments? n (%)		
Yes	26 (52)	18 (47)
No	24 (48)	20 (53)
Have you missed an oncology appointment due to lack of transportation? n (%)		
Never	44 (86)	32 (84)
Sometimes	6 (12)	5 (13)
Often	1 (2)	1 (3)
Have you delayed seeking medical care due to financial toxicities? n (%)		
Yes	7 (15)	10 (26)
No	39 (85)	28 (74)
Which mode of transportation do you use to come to medical appointments? n (%)		
Personal	25 (53)	19 (53)
Self-paid transportation	5 (11)	5 (14)
Insurance transportation	6 (13)	6 (17)
Public transportation	11 (23)	6 (17)
Distance from treatment clinic (miles)		
Median (range)	5.8 (0‐131)	5.8 (0‐217.1)
How do you feel about telephone or video visits in addition to in-person visits? n (%)		
I feel comfortable with phone/video visits	27 (53)	14 (40)
I prefer phone visits to video visits due to technological challenges	3 (6)	2 (3)
I always prefer in-person visits	21 (41)	22 (57)
Have you missed doses or delayed treatments due to not being able to pay for medications? n (%)		
Yes	6 (12)	5 (14)
No	45 (88)	32 (86)
Have you missed an oncology appointment due to caregiver issues? n (%)		
Never	47 (92)	34 (89)
Sometimes	2 (4)	3 (8)
Often	2 (4)	1 (3)
Evaluated by a social worker, n (%)		
Yes	40 (78)	38 (100)
No	11 (22)	0 (0)

Regarding transportation, the median distance from patients’ homes to the treatment center was 5.8 km in both groups. Although fewer than 20% missed appointments due to lack of transportation, many expressed concerns about the cost and accessibility of transportation to their medical appointments (ND group: 52% on day 0; CT group: 47% on day 0). Interestingly, many patients showed interest in telemedicine alongside in-person visits (ND group: 53% and CT group: 40% at day 0). We examined differences in social factors and subjective experiences of FT by ethnicity, and no statistical difference was found at day 0 in either cohort.

## Discussion

### Principal Findings

Our pilot study highlights a high prevalence of FT within the study population, affecting over 70% of participants. While no significant differences in FT were observed between patients ND and those undergoing CT, those in the CT group had significantly worse FT at day 90 compared to baseline (day 0). Race and ethnicity emerged as significant predictors of FT, with African American and Hispanic patients experiencing greater FT compared to non-Hispanic White counterparts. Although most patients could attend their oncology appointments and receive treatment, over one-third expressed concerns regarding access to transportation and associated costs.

### Comparison to Prior Work

Our findings are consistent with a growing body of literature that identifies FT as a persistent and complex challenge in cancer care [14]. Our patients in the CT group experienced worse FT over time, similar to a previously published study on CAR-T therapy [15]. The study by Maziarz et al [16] on health care costs in patients receiving allogeneic transplants reported that the median cost of health care for a patient receiving a transplant was over US $300,000. Apart from the high cost of SCT and CAR-T, prolonged hospital stays and multiple hospitalizations from treatment-related toxicities add to the burden of FT in these patients [17,18].

In addition to direct medical costs, indirect expenses—such as transportation, loss of employment from poor productivity due to illness, or switching to low-income jobs because they are less physically demanding—also add to the burden of FT [19,20]. Our study confirms these findings. In our study, although most patients made their oncology appointments, over a third were worried about access and the cost of transportation to attend them. Patients undergoing scheduled chemotherapy infusions need to make multiple visits to the clinic, which can be burdensome, especially for those in the CT group who must travel long distances to a FACT (Foundation for Accreditation of Cellular Therapy)-accredited center. This increases gas and public transportation costs, ultimately increasing the overall treatment cost burden [21,22]. Insurance coverage, while protective, may be insufficient to offset the rising costs. Increased deductibles, co-payments, and coinsurance have shifted much of the burden to patients, leading to substantial out-of-pocket expenses for their cancer care. Our study did not assess these out-of-pocket costs directly, nor did it quantify medical debt, areas that merit further investigation.

We found that race and ethnicity are significant predictors of FT, with African American and Hispanic patients experiencing worse FT than non-Hispanic White patients. Our results do not differ from studies on race and cancer-related FT in the literature [23-25]. Most of our patients are essentially racial and ethnic minorities and immigrants, and some are undocumented. They are likely to have lower incomes compared to their White counterparts, to be in the lowest socioeconomic tier, and have access only to public health insurance with limited coverage or even be uninsured [26-28]. Additionally, these patients are likely to have lower health literacy, leading to late disease presentation, increasing the intensity and cost of treatment. These findings underscore the structural inequities that contribute to FT among racial and ethnic minorities and immigrant populations. The convergence of social, economic, and health care–related factors amplifies financial strain and perpetuates disparities in outcomes. Addressing FT in these populations will require more than individual-level solutions; it demands systems-level change.

Beyond the direct effects of FT, it also encompasses the subjective financial distress—the emotional and psychological toll of economic strain [2,29]. This includes the depletion of household wealth and nonmedical budgets, as well as worries about the effectiveness of coping strategies [30]. In our study, over half of the patients experienced financial difficulty in paying for basic needs such as food, and more than 50% experienced emotional distress in their daily life, aligning with findings from Yu et al [31]. Although our results indicate a substantial emotional burden, further research is needed to determine whether this stems from receiving devastating news of a cancer diagnosis, the financial burden of treatment, or a combination of both. In our study, where over 50% of patients are unemployed, most of them will likely have to adjust their monthly budget and spend less on basic needs to pay for their cancer treatment. As their cancer treatment progresses, which is often prolonged in HM, they may face increasing financial burdens, leading them to adopt coping strategies such as relying on retirement savings, selling valuables, or borrowing from friends, family, or financial institutions [32]. In some cases, patients may resort to maladaptive strategies such as missing hospital appointments, medication nonadherence, or even stopping treatment entirely [33]. In our study, though a few patients missed or delayed treatments due to financial constraints, we see a decrease in the number of patients when followed up on day 30 and day 90. Although only a few patients in our study reported missing or delaying treatment due to financial constraints, we observed a decline in patient follow-up at 30 and 90 days. While the reasons for this attrition are not fully elucidated, the inability to afford ongoing treatment remains a plausible factor. These strategies may temporarily mitigate the financial impact of cancer care but ultimately lead to reduced quality of life, emotional distress, and devastating clinical outcomes.

### Limitations

Our study certainly has limitations. First, we had a low sample size, especially at the 90-day time point, as many of our patients were lost to follow-up, deceased, or chose not to continue with the study. Second, although our study was observational, the follow-up period may not have been long enough to detect a significant change in FT across time. Third, we did not collect data on the participants’ monthly income and the number of cycles of chemotherapy received at each survey time. This may have provided additional insights into assessing the severity of FT in our patient population. With our pilot study, we aimed to capture some of the barriers to cancer care in a unique patient population.

### Potential Solutions and Future Directions

Mitigating the burden of FT will involve developing and implementing intervention strategies at multiple levels, from the state/national level to the health insurance/payer, hospital, and provider levels [34]. In our study, many patients were worried about the transportation cost to their oncology appointments but were open to telemedicine visits. Therefore, incorporating telemedicine visits as an option in the care of our patient population could offer more flexibility for patients, reducing the travel burden and lost income from missed work [35]. Most of our patients were assessed by a social worker. While they play a role in identifying patients at high risk of financial distress, their assistance may not be sufficient, and they may not have the expertise to provide solutions to mitigate the severity of FT. A dedicated financial navigator, especially in a quaternary academic medical center, is required to help patients understand the economic aspects of their cancer care, budget appropriately, and maximize their employment and disability benefits in the context of ongoing financial commitment [36]. A larger cancer center–wide study is underway to evaluate the social determinants of health and better understand their implications on patient outcomes.

### Conclusions

This study highlights the significant and far-reaching impact of FT experienced by patients with cancer, particularly those from socioeconomically disadvantaged and ethnic and racial minority backgrounds. Our findings underscore the need for early FT screening and multilevel interventions to protect vulnerable populations from economic harm during cancer care.

## Abbreviations

CAR-T: chimeric antigen receptor T-cell therapy
COST-FACIT: Comprehensive Score for Financial Toxicity–Functional Assessment of Chronic Illness Therapy
CT: cellular therapy
FACT: Foundation for Accreditation of Cellular Therapy
FT: financial toxicity
HM: hematologic malignancies
ND: newly diagnosed
SCT: stem cell transplantation

## References

[1] Siegel RL, Miller KD, Fuchs HE, Jemal A (2022). Cancer statistics, 2022. CA Cancer J Clin.

[2] Zafar SY, Peppercorn JM, Schrag D (2013). The financial toxicity of cancer treatment: a pilot study assessing out-of-pocket expenses and the insured cancer patient’s experience. Oncologist.

[3] Pitis A, Diamantopoulou M, Panagiotou A, Papageorgiou D, Tzavella F (2025). Financial toxicity and its association with the quality of life of Greek patients with cancer: a cross-sectional study. Nurs Rep.

[4] McLean L, Hong W, McLachlan SA (2021). Financial toxicity in patients with cancer attending a public Australian tertiary hospital: a pilot study. Asia Pac J Clin Oncol.

[5] Oshiro NN, Nogueira L de A, Santos YHD, Guimarães PRB, Kalinke LP (2023). Quality of life and financial toxicity of hematopoietic stem cell transplant recipients in COVID-19. Rev Lat Am Enfermagem.

[6] Fitch K, Ferro C, Pittinger S (2019). The cost burden of blood cancer care in medicare. https://us.milliman.com/en/insight/the-cost-burden-of-blood-cancer-care-in-medicare.

[7] Bestvina CM, Zullig LL, Yousuf Zafar S (2014). The implications of out-of-pocket cost of cancer treatment in the USA: a critical appraisal of the literature. Future Oncol.

[8] Dieguez G, Ferro C, Rotter D (2018). The cost burden of blood cancer care. https://us.milliman.com/en/insight/the-cost-burden-of-blood-cancer-care.

[9] Ouchveridze E, Banerjee R, Desai A (2022). Financial toxicity in hematological malignancies: a systematic review. Blood Cancer J.

[10] Gordon LG, Merollini KMD, Lowe A, Chan RJ (2017). A systematic review of financial toxicity among cancer survivors: we can’t pay the co-pay. Patient.

[11] Lentz R, Benson AB, Kircher S (2019). Financial toxicity in cancer care: prevalence, causes, consequences, and reduction strategies. J Surg Oncol.

[12] The Bronx neighborhood profile. NYU Furman Center.

[13] de Souza JA, Yap BJ, Wroblewski K (2017). Measuring financial toxicity as a clinically relevant patient-reported outcome: the validation of the Comprehensive Score for Financial Toxicity (COST). Cancer.

[14] Jain T, Litzow MR (2018). No free rides: management of toxicities of novel immunotherapies in ALL, including financial. Blood Adv.

[15] Cusatis R, Tan I, Piehowski C (2021). Worsening financial toxicity among patients receiving chimeric antigen receptor t-cell (CAR-T) therapy: a mixed methods longitudinal study. Blood.

[16] Maziarz RT, Gergis U, Edwards ML (2024). Health care costs among patients with hematologic malignancies receiving allogeneic transplants: a US payer perspective. Blood Adv.

[17] McDiarmid S, Hutton B, Atkins H (2010). Performing allogeneic and autologous hematopoietic SCT in the outpatient setting: effects on infectious complications and early transplant outcomes. Bone Marrow Transplant.

[18] Ringdén O, Remberger M, Holmberg K (2013). Many days at home during neutropenia after allogeneic hematopoietic stem cell transplantation correlates with low incidence of acute graft-versus-host disease. Biol Blood Marrow Transplant.

[19] Abrams HR, Durbin S, Huang CX (2021). Financial toxicity in cancer care: origins, impact, and solutions. Transl Behav Med.

[20] Ehsan AN, Wu CA, Minasian A (2023). Financial toxicity among patients with breast cancer worldwide. JAMA Netw Open.

[21] Ambroggi M, Biasini C, Del Giovane C, Fornari F, Cavanna L (2015). Distance as a barrier to cancer diagnosis and treatment: review of the literature. Oncologist.

[22] Wercholuk AN, Parikh AA, Snyder RA (2022). The road less traveled: transportation barriers to cancer care delivery in the rural patient population. JCO Oncol Pract.

[23] Knight TG, Deal AM, Dusetzina SB (2018). Financial toxicity in adults with cancer: adverse outcomes and noncompliance. J Oncol Pract.

[24] Panzone J, Welch C, Morgans A (2022). Association of race with cancer-related financial toxicity. JCO Oncol Pract.

[25] Yabroff KR, Dowling EC, Guy GP (2016). Financial hardship associated with cancer in the United States: findings from a population-based sample of adult cancer survivors. J Clin Oncol.

[26] Pisu M, Kenzik KM, Oster RA (2015). Economic hardship of minority and non-minority cancer survivors 1 year after diagnosis: another long-term effect of cancer?. Cancer.

[27] (2021). Bronx borough health equity report 2021. County B.

[28] Frieden TR (2007). Health care access among adults in New York city. The importance of having insurance and a regular care provider. https://www.commonwealthfund.org/sites/default/files/documents/___media_files_resources_2007_new_report_examines_new_yorkers_access_to_care_access_dohmh_for_web_pdf.pdf.

[29] Zafar SY, Abernethy AP (2013). Financial toxicity, Part I: a new name for a growing problem. Oncology (Williston Park).

[30] Davis MP (2018). The MASCC Textbook of Cancer Supportive Care and Survivorship.

[31] Yu H, Li H, Zuo T (2022). Financial toxicity and psychological distress in adults with cancer: a treatment-based analysis. Asia Pac J Oncol Nurs.

[32] Carrera PM, Kantarjian HM, Blinder VS (2018). The financial burden and distress of patients with cancer: understanding and stepping-up action on the financial toxicity of cancer treatment. CA Cancer J Clin.

[33] Altice CK, Banegas MP, Tucker-Seeley RD, Yabroff KR (2017). Financial hardships experienced by cancer survivors: a systematic review. J Natl Cancer Inst.

[34] Yabroff KR, Bradley C, Shih YCT (2020). Understanding financial hardship among cancer survivors in the United States: strategies for prevention and mitigation. J Clin Oncol.

[35] Patel KB, Turner K, Alishahi Tabriz A (2023). Estimated indirect cost savings of using telehealth among nonelderly patients with cancer. JAMA Netw Open.

[36] Offodile AC, Gallagher K, Angove R, Tucker-Seeley RD, Balch A, Shankaran V (2022). Financial navigation in cancer care delivery: state of the evidence, opportunities for research, and future directions. J Clin Oncol.

